# Cutaneous cryptococcosis evaluation on reflectance confocal microscopy

**DOI:** 10.1016/j.jdcr.2024.10.027

**Published:** 2024-11-17

**Authors:** Alyssa Swearingen, Arianna Strome, Camila Ortiz, Shadi Khalil, Ata S. Moshiri, Amanda Levine

**Affiliations:** a Ronald O. Perelman Department of Dermatology, New York University Grossman School of Medicine, New York, New York; b Rutgers New Jersey Medical School, Newark, New Jersey

**Keywords:** cutaneous cryptococcosis, encapsulated yeast forms, infectious disease, reflectance confocal microscopy

## Background

In the United States, the incidence of cryptococcosis is estimated to be 0.4 to 1.3 cases per 100,000 individuals annually, with higher prevalence among immunocompromised patients.^1^ In organ transplant recipients, it is the third most common invasive fungal infection.^2^

Skin manifestations are often indicative of disseminated disease, including involvement of the pulmonary and central nervous systems. However, primary cutaneous lesions can also occur, although less frequently.^3^ Delays in diagnosis can lead to worse prognosis and fatal outcomes, making early detection crucial for disease control. Dermatologists are in an unusual position for prompt diagnosis of this condition, as many patients will initially seek care for skin lesions.

Herein, we report a rare case of a patient presenting with cutaneous cryptococcosis and subsequent examination on reflectance confocal microscopy (RCM). Our findings offer a discussion on the notable features found on confocal, which may aid in rapid clinical diagnosis.

## Case report

A 69-year-old woman presented to the dermatology clinic with a 2-month history of a painful pink papule on the right cheek. Her medical history was significant for renal transplant secondary to diabetic nephropathy, later complicated by microangiopathic hemolytic anemia. Her medications included belatacept monthly infusions, mycophenolate 500 mg twice daily, and prednisone 5 mg once daily for immunosuppression and ravulizumab for management of microangiopathic hemolytic anemia. The patient reported that the lesion first developed after cooking oil splashed on her face. She applied mupirocin ointment and clindamycin solution to the lesion for 1 month but noted no improvement with topical therapy.

On clinical examination of the right cheek, there was an erythematous, well-circumscribed papule with central ulceration measuring 1.0 × 0.8 cm. A small erythematous erosion was also present on the philtrum during the initial visit. Biopsy was deferred because of patient preference, and she was started on tacrolimus 0.1% ointment twice daily and continued on the mupirocin ointment twice daily. Patient was recommended to closely follow-up with our clinic in 1 month for reconsideration of biopsy.

**Fig 1:**
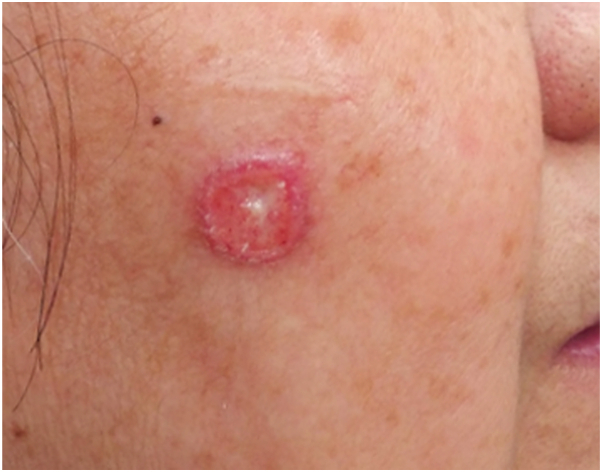
Cutaneous cryptococcal lesion on the right cheek.

**Fig 2:**
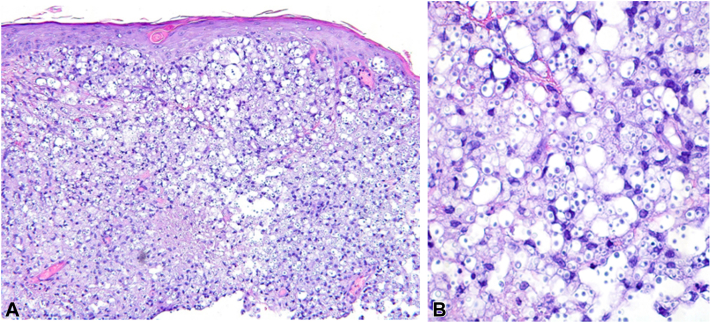
A, Histopathology (hematoxylin-eosin stain; original magnification: ×40) of the right cheek reveals granulomatous inflammation filling the dermis. Vacuolated spaces are readily apparent. **B**, Higher power; original magnification: ×200 demonstrates innumerable encapsulated yeast forms.

The patient returned to the clinic 1 month later without improvement of the right cheek lesion but the erosion on the philtrum had resolved. The central erosion of the cheek lesion had worsened, although overall size was approximately the same (Fig 1). Patient was agreeable to a biopsy at this time. Histopathology evaluation revealed focal ulceration of the epidermis overlying a dense granulomatous and mixed infiltrate extending from the papillary dermis to the base of the biopsy specimen. Innumerable yeast forms were seen admixed with inflammatory cells, many of which were surrounded by gelatinous capsules (Fig 2, *A*, *B*). Histopathology findings confirmed the diagnosis of cutaneous cryptococcosis.

**Fig 3:**
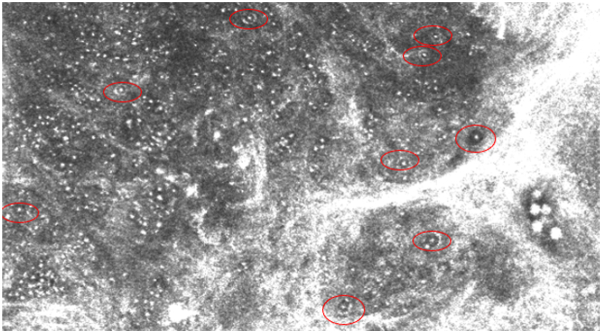
Reflectance confocal microscopy imaging. Select yeast forms circled in red.

Two weeks after the pathology results, the patient returned to the clinic to discuss the next steps in management. The patient denied fever, chills, cough, shortness of breath, chest pain, and vision changes. The lesion had become further eroded. On dermatoscopy, larger dotted vessels were seen in the central, ulcerated region and comma vessels along the border of the lesion. At this time, confocal microscopy was performed for research purposes. Imaging was completed using the handheld VivaScope 3000 (Caliber ID) device along the perimeter of the lesion. RCM imaging revealed the presence of diffuse inflammatory cells and encapsulated yeast forms (Fig 3).

The patient was evaluated by infectious disease and was started on oral fluconazole 200 mg twice daily, until central nervous system involvement was assessed. Cryptococcal serum antigen was elevated at 160 (ref <2). A lumbar puncture was performed, and cerebrospinal fluid analysis revealed a negative fungal culture, India ink stain, and cryptococcal antigen. The meningitis-encephalitis pathogen panel by polymerase chain reaction was also unremarkable. The patient returned to the dermatology clinic 3 weeks after starting fluconazole with noticeable improvement in her cutaneous lesion.

## Conclusion

This case presents a rare report of cutaneous cryptococcus visualized on RCM and confirms that encapsulated yeast forms are visible on imaging. Cryptococcus neoformans is known to incorporate melanin into its cell wall, which provides protection from environmental stressors and is associated with increased virulence.^4^ We hypothesize that the presence of melanin in the fungal cell wall may account for the visibility of yeast forms, as the reflective nature of melanin on RCM results in a brighter appearance. Further research is needed to definitively elucidate the mechanism underlying the visibility of these yeast forms. Nevertheless, using RCM during clinical evaluation allows for the detection of yeast and differentiation from other skin conditions, which can facilitate rapid diagnosis and improve patient outcomes.

## Conflicts of interest

None disclosed.
